# Precision Medicine: An Emerging Paradigm for Improved Diagnosis and Safe Therapy in Pediatric Oncology

**DOI:** 10.7759/cureus.16489

**Published:** 2021-07-19

**Authors:** Puneet Singla, Pankaj Musyuni, Geeta Aggarwal, Harjinder Singh

**Affiliations:** 1 Pediatrics, Government Medical College, Patiala, IND; 2 Pharmaceutics, Delhi Pharmaceutical Sciences and Research University, New Delhi, IND

**Keywords:** genomic diagnosis, clonal evolution, precision cancer medicine, network analysis, pediatric oncology, research and innovation

## Abstract

Cancer is a lethal disease that kills a great number of people each year. Standard treatments such as chemotherapy or radiation are only effective in a small percentage of individuals due to illness variability. Tumors can be caused by a variety of genetic factors and express a variety of proteins depending on the individual. Because of developments in high-throughput technology, there has been a flood of large-scale biological data produced in recent decades. As a result, the focus of medical research has evolved. It was a once-in-a-lifetime chance for translational research to explore molecular alterations across the entire genome. In this setting, precision medicine was developed, and the possibility of better diagnostic and treatment tools became a reality. This is especially true in the case of cancer, which is becoming more prevalent around the world. The goal of this study is to look at precision medicine technology and its applications to cancer, with a focus on children. The inherent diversity of cancer lends itself to the rapidly expanding field of precision and personalized medicine.

## Introduction and background

Cancer, though a broad term, refers to the uncontrolled proliferation of cells that are prone to stochastic somatic mutations in oncogenes and tumor suppressor genes. Both the intrinsic fragile genomic integrity and inheritance of DNA-damaging genes, as well as exogenous oncogenic mutations fueled by infections and varied environmental factors, are implicated in the acquisition of cancer in children, according to cell biologists. Around 400,000 kids and young people between the ages of 0 and 19 are diagnosed with cancer each year around the world [1]. Cure rates differ substantially between high-income nations (about 80%) and low- and middle-income nations (about 50%). Even though cure rates have risen from 20% to 80% in the last 50 years, owing primarily to risk stratification and the implementation of multimodality treatment methods, it is still critical that we use today's advanced technology to develop more effective and less toxic treatment options for children with cancer. At the genetic level, variations between the cancer kinds in patients persist due to continuing mutations, rendering generic treatments ineffective. Every person's genome contains small single-nucleotide polymorphisms (SNPs) and/or significant alterations in the DNA base pair sequence (mutations). These can be inherited, but exogenous factors can also introduce them into a person's life over the course of one's lifetime (e.g., carcinogenic chemicals or radiation). Even though these genetic changes are usually harmless to an individual's health, they can affect how the body responds to a therapeutic substance, either through changes in the drug target or through adverse drug-drug interactions with absorption, distribution, metabolism, excretion, and toxicology (ADMET) concerns [2].

Cancer is responsible for one out of every six deaths, according to a World Health Organization (WHO) report. In addition, the poll revealed that cancer claimed the lives of nearly 9.6 million people in 2018. Lung (2.09 million cases), breast (2.09 million cases), colon and rectum (1.80 million cases), prostate (1.28 million cases), skin (1.04 million cases), and stomach (1.03 million cases) are the most affected organs, while lung, colorectal, stomach, liver, and breast cancers are the most common causes for cancer death and account for significant morbidity [3].

According to a report published by the American Cancer Society (ACS), there will be 1,898,160 new cancer cases and cancer will claim the lives of 608,570 people in 2021. The report went on to say that there are 5200 new cases and 1670 cancer deaths every day in the United States of America. Furthermore, the National Cancer Institute (NCI) predicts that new cancer cases will primarily be affected by cancer of breast and lung, the prostate, colon and rectum, skin, and bladder, non-Hodgkin lymphoma, cancer of kidney and renal pelvis, endometrial cancer, leukemia, pancreatic, thyroid, and liver cancer, among others. Prostate, lung, and colorectal cancers are the most common cancers in men, accounting for approximately 43% of all cancers in the population, according to the study's findings. Breast, lung, and colorectal cancers are the leading cause of cancer deaths. New cancer cases and deaths are estimated to occur at a rate of 442 cases and 158 deaths per 100,000 people, respectively, according to the study, with mortality being higher in men, who account for approximately 189 and 135 cases per 100,000 people. It is predicted that the number of cancer cases worldwide will increase from 18,078,957 to 29,532,994 million between 2018 and 2040, according to a graphic tool developed by the International Agency for Research on Cancer. It also predicted a rise in mortality from 9,555,027 to 16,388,459 cases by 2040, with an increase of more than 50% [4,5].

Precision medicine, also known as personalized medicine or PM, refers to therapies, such as therapeutic agents, that are tailored to specific patients or groups of patients. PM is a subset of personalized medicine, and vice versa. The primary goal is to match therapies to individuals to provide the most effective treatment with the fewest side effects. This is extremely crucial for cancer sufferers, who only have a few months to live after being diagnosed. Furthermore, the cost of sequencing the human genome has steadily decreased, resulting in the widespread use of integrated sequencing technologies, as well as the PM method, in recent years. Although the terms "personalized medicine" and "precision medicine" are frequently used interchangeably, the term "precision medicine" was coined by the United States National Research Council in 2011 to convey a wider definition that, while it may not be possible to develop treatments explicitly for individual patients at an individual level, it should be possible to define subsets of patients and target them with treatments. When the PM initiative was launched in the United States in 2015, it gave a significant boost to the concept of precision medicine in oncology, as well as accelerated the development of biomarker-driven treatment methods [6,7].

The most essential aspect of a PM strategy in oncology is the recognition of a "biomarker" related to a specific cancer type. A biomarker is a mutated nucleic acid sequence, protein, glycoprotein, or set of proteins that is expressed solely by tumor cells and not by healthy cells, according to the NCI. Biomarkers are classified into four types: predictive (which cohort of patients may benefit from a specific drug therapy), diagnostic (which confirms that the patient has a specific cancer), predictive (which suggests how the cancer may develop in the individual), and prognostic (which suggests how the cancer may develop in the individual). The NCI defines a biomarker as "a biological molecule found in blood, other bodily fluids, or tissues that is a sign of a normal or abnormal process, or of a condition or disease" [8,9]. The biomarker is useful in determining the response to a specific therapy administered to cancer sufferers. The "-omics" technologies have been widely used for identifying biomarkers, and the tests include the collection of biological specimens such as biopsy tissues, patients' blood, and/or urine samples. Furthermore, a pre-clinical assay has indeed been performed, and clinical verification is underway. Table 1 lists some illustrations of precision medicine being used in various types of cancer [10-12].

**Table 1:** Examples of precision medicine biomarkers in cancer HER2: human epidermal growth factor receptor 2; BCR-ABL: a predictive biomarker for use of dasatinib, bosutinib, nilotinib, imatinib, and ponatinib; BRAF: v-raf murine sarcoma viral oncogene homolog B1; EGFR: epidermal growth factor receptor; ALK: anaplastic lymphoma kinase

Biomarker/drug	Cancer
HER-2/neu receptor/Herceptin	Breast cancer
BCR-ABL/ Gleevec	Chronic myeloid leukemia
BRAFV600E/Zelboraf	Melanoma
EFGR/Erbitux	Colon and related cancer
ALK/Xalkori	Lung cancer

## Review

Precision medicine

PM can be described in multiple ways. The application of new molecular data analysis to better manage a patient's illness or genetic disposition to disease, for example, is defined by the Personalized Medicine Coalition (PMC) as "the use of new methods of molecular analysis for better management of a patient's disease or predisposition to disease." PM, on the other hand, is defined by the American Medical Association as "health care informed by each person's unique clinical, genetic, and environmental information." The NCI has a different definition that includes both prevention and diagnosis. PM is defined as "a type of medicine that prevents, diagnoses, and treats diseases using information about a person's genes, proteins, and the environment" [13].

According to the President's Council of Advisors on Science and Technology in the United States, precision medicine is defined as "the tailoring of medical treatment to the individual characteristics of each patient and the ability to classify individuals into subpopulations that differ in their susceptibility to a particular disease or their response to a specific treatment." Though there is a fine line between the terms "personalized medicine" and "precision medicine," the latter has surpassed the former since it considers a wider range of a patient's biological data, such as clinical characteristics, lifestyle behaviors, and genetic composition. Precision medicine suggests the novel notion of using an individual's biomarker data to generate highly specific diagnosis and treatment regimens, which goes beyond the traditional "signs-and-symptoms" approach. Precision medicine, as opposed to the ineffective "one patient, one pill" approach, separates the population into subgroups based on genetic patterns, environmental impacts, and pharmacological reactions, and then develops a unique therapy strategy for each subgroup. This concept has been realized in the form of "The Knowledge Network of Disease," a new taxonomy system [14].

**Figure 1:**
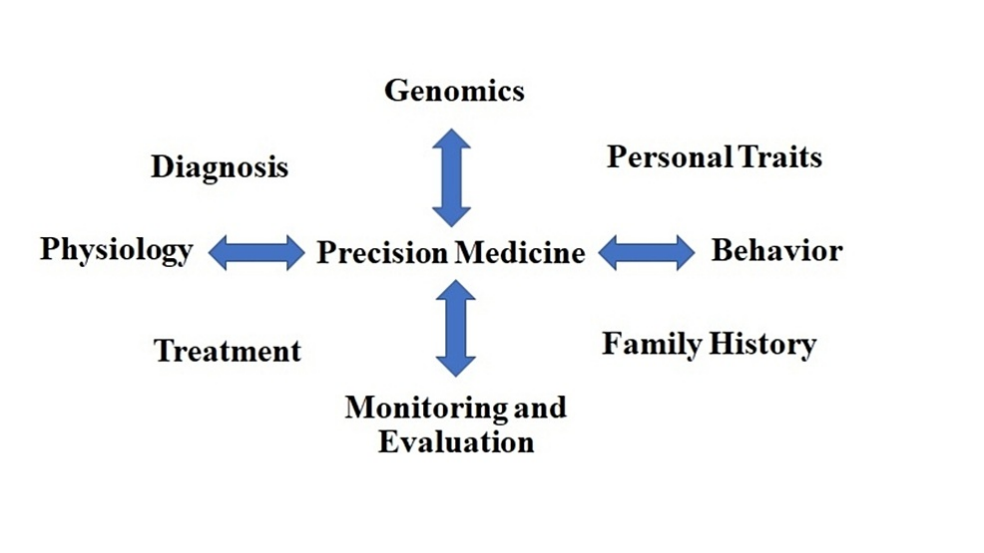
Tools of precision medicine strategy

Precision medicine is a cutting-edge technique for addressing the faults in traditional cancer treatment. This method is based on manipulating patient genetic and -omic data (genomics, transcriptomics, metabolomics, and proteomics). The current research examines this novel method and its potential to revolutionize pediatric oncology. The FDA has approved monoclonal antibodies, checkpoint inhibitors, and adoptive cell therapy for use in children, whereas vaccines and oncolytic virotherapy are currently being studied to see if they can treat children with cancer. PM can be thought of as a tool for preventing and treating health problems in everyday life. The element of PM in cancer treatment relates to genomic or molecular testing, targeted therapies, and the use of genomic markers. Genomic testing usually refers to providing improved testing for biological samples with the goal of providing a cost-effective solution. Because cancer is caused by the gradual accumulation of mutations in genes, genomic testing is the most important factor in analysis [15,16]. Precision medicine is a method of using genomic information to improve cancer diagnosis and treatment options that are personalized to the tumors of individuals. Drugs have been produced to combat cancer in a variety of ways because of studies into the genetic abnormalities linked to the disease by inhibiting enzymes that cause cancer cells to proliferate and survive abnormally, blocking the abnormal gene expression found in cancer cells, and stopping the overactive molecular signaling pathways in cancer cells. These "targeted medicines" are designed to address cancer cells' distinct properties from normal bodily cells. As a result, they are less likely to be hazardous to patients than other treatments that might harm normal cells, such as chemotherapy and radiation. Figure 1 depicts the tools used in PM for providing a better assessment.

The method used in the testing involves comparing the DNA of tumor cells to that of normal cells. PM eventually assists in recommending the optimal dosage as well as precautions such as diet and toxin exposure. The approach can be understood by using the surface protein KRAS for colon cancer and the targeted options "trastuzumab" and "nivolumab." Targeted therapies, on the other hand, work through a mechanism that promotes angiogenesis or tumor cell progression. Monoclonal antibodies and small-molecule drugs are two types of drugs in this category. Furthermore, the elements of genomic care, which employ the steps of diagnostic testing, monitoring progression, guiding treatment, and assessing response, are critical [17-19]. Table 2 lists examples of FDA-approved monoclonal antibodies for cancer as well as examples of targeted drugs [20,21].

**Table 2:** List of some FDA-approved precision medicines for treating cancer HER2: human epidermal growth factor receptor 2; EGFR: epidermal growth factor receptor; VEGF-A: vascular endothelial growth factor A; PD-1: programmed cell death protein 1; PD-L1: programmed death ligand 1; mTOR: mammalian target of rapamycin; PARP: poly(ADP-ribose) polymerase

FDA-approved precision medicine	
Monoclonal antibodies used as a precision medicine	
Trastuzumab	HER2/breast cancer
Cetuximab and panitumumab	EGFR/colorectal cancer
Bevacizumab	VEGF-A/colorectal cancer
Pertuzumab and trastuzumab emtansine	HER2/breast cancer
Nivolumab and pembrolizumab	PD-1/melanoma, non-small-cell lung cancer
Necitumumab	EGFR/non-small-cell lung cancer
Atezolizumab, durvalumab, and cemiplimab	PD-L1/bladder cancer and cutaneous squamous cell carcinoma
Examples of some targeted drugs useful as a precision medicine	
Molecules	Axitinib, bevacizumab, sunitinib
Molecular target and indication	"Angiogenesis"; axitinib is approved for renal cell carcinoma, bevacizumab is approved for metastatic colon, lung, renal, ovarian, GBM, and sunitinib for renal cell carcinoma, GIST and target is VEGF
Molecules	Traxtuzumab and pertuzumab
Molecular target and indication	Acting via signal transduction and approved for breast cancer and target is HER2
Molecules	Bortezomib and carfilzomib
Molecular target and indication	"Apoptosis," and approved for myeloma, mantel cell lymphoma having target for proteasome
Molecules	Olaparib
Molecular target and indication	"DNA repair" and approved for ovarian cancer targeting PARP
Molecules	Temsirolimus
Molecular target and indication	Using biological target of protein synthesis, approved for breast and renal cell carcinoma targeting mTOR

Precision medicine in pediatric oncology

For molecular precision analysis, numerous methods can be used. Whole-exome sequencing (WES), whole-genome sequencing (WGS), and RNA sequencing can be done independently or in conjunction with methylation and expression microarray analysis. DNA methylation-based molecular diagnostics are now being used to classify pediatric central nervous system tumors. A growing number of gene-based targeted sequencing panels are now commercially available for use in clinical practice. When compared to WGS or WES studies, targeted sequencing improves the ability to detect low-level clonal variants within a tumor.

**Figure 2:**
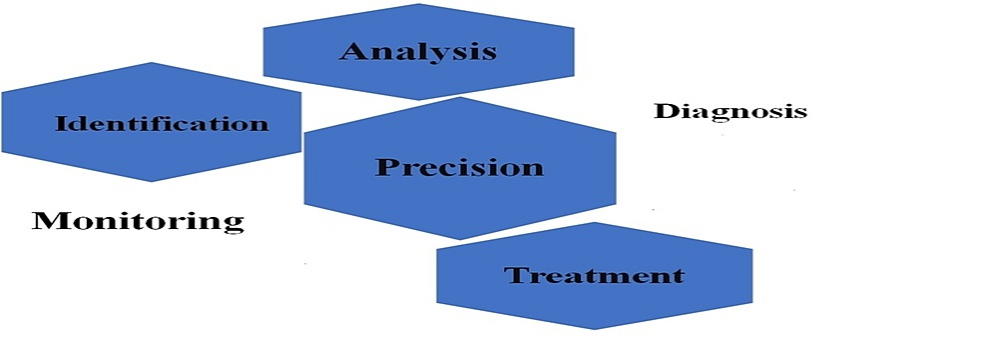
Sequencing of tumor analysis and treatment

Although mutational testing for all pediatric tumors has been advocated, it is also critical to identify patient populations that may benefit the most from molecularly targeted therapy and accelerated development of novel drugs for early phase clinical trials. The most effective clinical and realistic applications of tumor sequencing can be seen in the following scenarios (Figure 2).

Role of PM in treating cancer

PM has the potential to shift the focus of medicine from treatment to prevention. This would be possible if we could somehow predict who might develop the disease, allowing patients to benefit from early preventative treatment. It also aids in proper treatment selection: this can be accomplished by reducing trial and error prescribing, which delays the start of intensive treatment; this, in turn, leads to a high level of medication adherence. PM also seeks to achieve its goals while avoiding therapy delays, which would undoubtedly improve quality of life and is one of the treatment's main goals with cost-effective medication. In the last few decades, cancer therapies have progressed at an extraordinary rate. Although the goal of any treatment option is to cure the disease, because of the resistant nature of cancer, this is rarely attainable depending on the tumor's kind, location, and advancement, as well as the patient's age and overall condition. A single or a mix of classical alternatives, surgery, radiation, chemotherapy, and the recently enhanced immunotherapy are used in traditional cancer treatment. Surgical excision of a localized tumor in an accessible part of the body is the most common use of surgery [22].

More advanced treatments, such as radiation and chemotherapy, are required for metastatic cancers. They use high doses of radiation and cytotoxic medicines to elicit an immune response that kills cancer cells and the microenvironment around them. However, these methods endanger quickly dividing healthy cells with a fast turnover, resulting in a slew of adverse effects and systemic toxicity. Opposing the "one size fits all" approach, immunotherapy has emerged as the forerunner of a more personalized, effective, and less toxic therapeutic option, establishing it as a clinically recognized cancer therapy. Table 3 lists some of the drugs that have been approved for the treatment of pediatric cancers [23,24].

**Table 3:** Drugs approved for childhood cancers

Type of cancer	Drugs
Colorectal cancer	Ipilimumab, nivolumab
Epithelioid sarcoma	Tazemetostat hydrobromide
Ewing sarcoma	Dactinomycin
Gestational trophoblastic disease	Dactinomycin
Giant cell astrocytoma	Everolimus
Hodgkin lymphoma	Pembrolizumab, procarbazine hydrochloride
Drugs approved for leukemia	
Acute lymphoblastic leukemia	Blinatumomab, clofarabine, cyclophosphamide, cytarabine, dasatinib, daunorubicin hydrochloride, imatinib mesylate, mercaptopurine, methotrexate sodium, nelarabin, vincristine sulfate
Acute myeloid leukemia	Gemtuzumab ozogamicin (Mylotarg), vincristine sulfate
Blastic plasmacytoid dendritic cell neoplasm	Tagraxofusp-erzs
Chronic myelogenous leukemia	Cytarabine, dasatinib, imatinib mesylate, nilotinib
Melanoma	Ipilimumab
Merkel cell carcinoma	Avelumab, pembrolizumab
Neuroblastoma	Dinutuximab, naxitamab-gqgk, vincristine sulfate
Neurofibromatosis type 1	Selumetinib sulfate
Non-Hodgkin lymphoma	Crizotinib, nelarabine, vincristine sulfate, pembrolizumab
Pheochromocytoma and paraganglioma	Iobenguane I-131
Rhabdomyosarcoma	Dactinomycin, vincristine sulfate
Wilms tumor and other childhood kidney cancers	Dactinomycin, doxorubicin hydrochloride, vincristine sulfate

PM trials

A PM trial, unlike a traditional oncology trial, is essentially an enrichment trial that uses an adaptive design to develop a gene agonistic-tumor specific clinical study, eventually leading to the discovery of the best-matched targeted medicine. Due to developments in pediatric oncology, these trials are continually evolving. Patients with a single histology tumor type are subdivided into genetic variants that are specifically matched to their anti-cancer medications in an umbrella research. A basket trial, on the other hand, focuses on a common genetic flaw that has been linked to a variety of cancers. The availability of an experimental treatment for a rare tumor with the same histological abnormalities as other typically occurring tumors could be a possible benefit of this technique. Platform studies use the same analytical technology, like next-generation sequencing (NGS), to find biomarkers in a variety of tumor types.

Diagnostic testing procedures and technologies

Tests are usually based on different technologies and are not limited to the specific examples mentioned herein. Immunohistochemistry (IHC) is a technique that uses antibodies and antigens to distinguish between abnormal (e.g., malignant) and healthy cells and tissues. Although IHC can effectively visualize the localization of various antigens and biomarkers, it has several limitations, the most significant of which is intra-observer variability in determining the concentration and quantity of true cellular counterstain [25]. In situ hybridisation (ISH) is another technique that employs fluorescent nucleic acid probes to recognise, bind to, identify, interpret, and track changes in genes or gene regions found only in tumor cells. Many of these tests are limited to detecting single specified alterations, whereas cancer cells can develop multiple mutations [26].

Immunoassays are antibody/antigen recognition tests that can identify a wide range of biomarkers, including those related to toxic effects, active drug metabolic products in the blood, and total drug levels for therapeutic tracking. Serum marker testing kits for various cancers may be non-specific [27]. Polymerase chain reaction (PCR) is a technique based on DNA amplification that can identify single-base changes within genes, making it a great choice for detecting gene-based tumor cells. However, if the test sample is deteriorated, the amount of error results may increase. Flow cytometry uses a laser or impedance-based biophysical technology to sort and count cells, and biomarker identity is managed to accomplish by passing a cell suspension through a digital sensing element. This allows for instant multiparametric analysis of chemical and physical properties of hundreds of cells in milliseconds. Flow cytometry saves cells for future studies, but it involves a large number with fluorophores to distinguish cell types, resulting in huge amounts of data that can be difficult to evaluate and understand. Tandem mass spectrometry, when combined with liquid, gas, as well as capillary chromatography methods, can be used to reliably assess the quantities of biomarkers and medicines in biological samples [28].

NGS is a high-throughput technology for screening short or long nucleotide sequences and identifying biomarkers that are highly expressed. Although technology is still very expensive, it is becoming more widely accepted as a means of diagnosing, forecasting risk, and classifying different types of cancer. For example, the FDA has issued two guidance letters to encourage the use of such NGS as a clinical diagnosis for determining the risk of having a genetic disorder. The first provides FDA-approved public databases containing patient information that can be used by CDx test makers to confirm the effectiveness of NGS-based genomic testing results. The second document advises prospective manufacturers on how to develop NGS testing, highlighting key components that the FDA will take into consideration for an assay's analytical validity in a pre-market application [29,30].

Current challenges in the path of precision medicine

Dealing with a vastly diverse and complicated enemy like cancer entails a wide range of obstacles and problems at practically every stage of execution. To ensure that the maximum number of patients benefit, an exceptionally sophisticated and well-equipped model is required, starting with policymakers, and ending with the beneficiary (a cancer-stricken patient). Precision medicine trials are difficult to execute because of the time-consuming process of matching patients to medications. This is primarily owing to the widespread use of tiny gene panels in many patients, which requires a long time to identify genetic changes that are truly treatable. Patients must meet severe qualifying requirements based on their comorbidities, which may lead to a misrepresentation of the actual patient population. Even if this occurs in a timely manner, limiting drug availability means that not all matched patients will receive targeted therapy.

Due to histological co-alterations, the effects of matched (or targeted) therapy vary with the patient. Sequencing genetic indicators specific to each patient's histological cancer type, which are predictive of therapy response, can help overcome this. We must also consider the fact that a targeted drug's pharmacodynamics and pharmacokinetics may differ between ethnic groups. Because the cost of omic and non-omic analysis has decreased, sequencing techniques have become more widely used, resulting in a massive volume of "big data." The urgent need is to create a standardized platform for integrating this data that can be used by healthcare practitioners to guide care. To advance the precision medicine agenda and broaden the horizon of medical treatment, fluid and transparent collaboration among all stakeholder groups is critical. It is a new, exciting era in medication, one that will unquestionably improve the quality of care given to patients, depending on the specific differences. Healthcare will be tailored to the patient's specific needs, ensuring that the most appropriate and effective medication is prescribed with the fewest complications and adverse effects.

Future prospects

The availability of precise diagnostics capable of identifying patients who would benefit from personalized therapy is crucial to the success of precision medicine. NGS technology has demonstrated a more molecular biological characterization of cancers, having contributed to the enormous commitment to precision medicine. Cancer gene panels based on NGS examine the physicochemical structure of tumor tissues all at once and could provide sufficient coverage to recognize minor allele frequencies in a cost-effective manner. A new clinical "basket trial" design ranging across multiple sites has shown some achievement in directing patients with rare tumors to the best possible treatments. Off-label drug use based on NGS data, as well as cancer therapy predicated on signaling pathways, has been successful. The use of cfDNA has given scientists renewed hope that they will be able to provide medications to patient populations at the right dose and at the correct time.

The enormous complexity of the cancer genome, on the other hand, indicates that we are still in the initial phases of interpreting molecular data and transforming it into an understanding that clinicians and cancer patients can use. So many cancer genomes must be studied to gain a better comprehension of tumors and develop new molecular analysis tools. More clinical trials using biochemical criteria in both adults and children are required. Medical applications of ctDNA are expected to grow in the coming years as prospective genotype-based tailored medications are developed. Finally, orthogonal technologies such as functional investigations, in relation to NGS-generated cancer information such as transcriptomes via RNA sequencing and high-resolution cancer methylomes, are required to accelerate the era of precision medicine in cancer. The most efficient use of all this data, combined with an integrative framework across cancer types, will result in a comprehensive molecular pathway that underpins tumor genesis and evolution. Consequently, every cancer sufferer will have access to a detailed map of molecular events.

## Conclusions

It is to be expected that healthcare difficulties and illness patterns vary by country and are influenced by a variety of factors. PM's approach to various conditions, including heart disease, diabetes, and neurological illnesses, may be expanding. Given the limitations, data requirements, and multiple steps involved in precision therapy, it is preferable to form public-private partnerships that can be extended to the precompetitive space between academia, industry, and government to aid in the promotion of research by providing necessary support. Improving reproducibility and sharing preclinical data may help to strengthen the drug development pipeline by raising standards and sharing preclinical data. Several factors may influence investment decisions, which researchers may explore to increase the likelihood of their drug's success. Furthermore, the utilization of technology such as artificial intelligence, machine learning, and a shift in business model would enable certain problems to be addressed, as well as the smoothing out of the stages involved in providing precision therapy. Innovative strategies are beneficial for disease regulation while they are being developed. On the other hand, having a justification will help with research orientation. PM's policy is likely to aid in the better utilization of existing pharmaceuticals as well as support ongoing oncology medication research. Precision medicine is predicted to generate good opportunities in the healthcare sector, as well as job opportunities.
